# MicroRNAs Regulate Bone Development and Regeneration

**DOI:** 10.3390/ijms16048227

**Published:** 2015-04-13

**Authors:** Sijie Fang, Yuan Deng, Ping Gu, Xianqun Fan

**Affiliations:** Department of Ophthalmology, Shanghai Ninth People’s Hospital, Shanghai JiaoTong University School of Medicine, Shanghai 200011, China; E-Mails: fangsijie89@hotmail.com (S.F.); ophden@163.com (Y.D.); guping2009@hotmail.com (P.G.)

**Keywords:** microRNA, osteogenesis, bone regeneration

## Abstract

MicroRNAs (miRNAs) are endogenous small noncoding ~22-nt RNAs, which have been reported to play a crucial role in maintaining bone development and metabolism. Osteogenesis originates from mesenchymal stem cells (MSCs) differentiating into mature osteoblasts and each period of bone formation is inseparable from the delicate regulation of various miRNAs. Of note, apprehending the sophisticated circuit between miRNAs and osteogenic homeostasis is of great value for artificial skeletal regeneration for severe bone defects. In this review, we highlight how different miRNAs interact with diverse osteo-related genes and endeavor to sketch the contours of potential manipulations of miRNA-modulated bone repair.

## 1. Introduction: Functions and Canonical Biogenesis of MiRNAs

Lin-4 is the first small RNA discovered in 1993 by Victor Ambros and his colleagues in nematode worms. Seven years later, the discovery of another lin-4-like RNA, let-7, opened up a world of these small noncoding RNAs [1] and studies focusing on miRNAs have been emerging in an endless stream since then. The miRBase Sequence Database published in the 20 June 2013 reported that 30,424 mature microRNAs (miRNAs) had been elucidated in 206 species, including plants, viruses, fruit flies, nematode worms, and mammals [2]. MiRNAs are endogenous ~22-nt RNAs that exert vital regulating functions on many fundamental physiological and pathological processes in multiple organisms via targeting messenger RNAs (mRNAs) for degradation or translational repression [3,4]. In mammals, miRNAs are detected in almost all cells, tissues and organs, which are involved in lots of pivotal bio-processes, including cell death and/or cell proliferation such as stem cell division and hematopoietic differentiation, developmental timing such as neuronal, muscle and heart development, substance metabolism, oxidative stress resistance, and so forth. They also modulate the balance between proto-oncogenes and tumor suppressor genes [1,5,6]. MiRNAs are single-stranded RNAs (ssRNAs) generated from endogenous hairpin-shaped transcripts. Approximately 50% of mammalian miRNA loci are found in close proximity to other miRNAs [1,7]. These clustered miRNAs are transcribed from a single polycistronic transcription unit (TU). The primary transcript (pri-miRNA) is formed by RNA polymerase II (Pol II), which is composed of a double stranded stem of ~33 base pairs, a terminal loop and two flanking unstructured single-stranded segments. The cleavage of pri-miRNA is then mediated by the RNAase III endonuclease Drosha in the nucleus, producing a 60~70-nt stem-loop intermediate, called the pre-miRNA, which is recognized and exported to the cytoplasm by the karyopherin XPO5 (exportin-5) upon a Ran-GTP-dependent manner. In the cytoplasm, a ~22-nt double-stranded RNA duplex termed miRNA duplex is released by cleavage via another RNAse III endonuclease named Dicer. Only one chain of the short RNA fragment is delivered to, or loaded onto, the RNA-induced silencing complex (RISC) containing an argonaut protein, whereas the other one is degraded. Finally, this mature complex controls specific gene expressions at the post-transcriptional level upon targeting 3'-untranslated regions (3'-UTRs) of mRNAs and initiating either their degradation or a reduction in translational efficiency [4,7,8,9].

Bone defects, caused by traumas, congenital malformations or diseases, are enormous social and economic burdens, which seriously affect people’s quality of life worldwide. As various miRNAs have been reported by many researches in recent years to regulate skeletal homeostasis and osteoblastogenesis either positively or negatively through multiple signaling pathways, this review focuses on the biological functions of miRNAs on bone formation and metabolism. It will shed light on the potential therapeutic strategies for bone loss by miRNA treatment and provide a novel look on artificial bone regeneration with miRNA-based methods.

## 2. Evidence Supporting MiRNAs Participating in Osteogenesis

Bone, a mineralized mesenchymal tissue, is not only a support against mechanical forces, but also an endocrine organ mainly modulating mineral homeostasis and energy metabolism [10]. During embryogenesis, bone formation is divided into two independent programs: intramembranous and endochondral ossification. In the former pathway, craniofacial bone is generated upon the differentiation of the condensed mesenchyme into osteoblasts, while the latter one facilitates long bone growth. The anabolic activities of osteoblasts and the catabolic actions of osteoclasts result in continuous self-renewal bone tissues in vertebrates, keeping an appropriate bone mass and maintaining calcium homeostasis [11,12,13]. Osteoblasts, deriving from multipotent mesenchymal stem cells (MSCs), have been verified to produce extracellular matrix (ECM) and make it mineralization, thus directly forming intramembranous bones. Furthermore, they affect osteoclast differentiation from hematopoietic cells as well [14,15]. There are three consecutive stages of osteoblastogenesis: proliferation/growth, matrix maturation, and mineralization/nodule formation, which are characterized by temporally and spatially expressed genes and are finely tuned by a host of miRNAs [16].

Analogous to some specific miRNAs highly expressing in different organs such as miR-183 clusters enriched in sensory organs [5], quite a few studies have already delineated various miRNAs that are peculiar to the bone tissues to a certain extent. MiR-138 is expressed in mouse calvaria, liver, heart, and brain and at a low level in long bones and bone marrow. It is absent in cartilage [17]. MiR-204 and miR-211 are detected in different mouse and human mesenchymal progenitor cell lines [18]. *In situ* hybridization exhibited a high expression level of miR-335-5p in osteoblasts and hypertrophic chondrocytes of mouse embryos [19]. Two consecutive studies from the same group reported that both miR-2861 and miR-3960 are highly expressed in primary mouse osteoblasts, lowly detected in liver and barely found in other mouse tissues and osteoclasts [20,21]. Additionally, both miR-140-5p and miR-140-3p are enriched in human mesenchymal stem cells (hMSCs) from several tissue sources, including human adipose-derived stem cells (hADSCs), human bone-marrow-derived stem cells (hBMSCs), and human umbilical cord-derived stem cells (hUCSCs) [22]. This is the first thing to concern, for it drops a hint that these bone tissue or MSC enriched miRNAs are possibly essential for osteogenesis, which will be further discussed below.

Moreover, Kobayashi *et al.* first reported that disruption of the *Dicer* gene governed by the collagen type II alpha 1 (COL2A1) promoter *in vivo* leads to severe bone growth defects and premature death of mice [23]. Soon afterwards, another elegant study by Gaur *et al.* further determined the functional role of miRNAs in skeletal development. *Dicer* was silenced in osteoblasts through the cre-recombinase expressed from the promoter of either the rat collagen type I alpha 1 (COL1A1) gene or the human bone gamma-carboxyglutamic acid-containing protein/osteocalcin (BGLAP/OC) gene. Their results demonstrated the irreplaceable position of *Dicer* in proper bone development and mineralization [24]. In agreement with what Gaur *et al.* found, Raaijmakers also showed that conditional ablation of *Dicer1* in mouse osteoprogenitors disrupts the integrity of hematopoiesis, leading to reduced bone marrow stromal osteogenic colony number with impaired osteogenic differentiation [25]. Altogether, these studies imply a pivotal role of miRNAs in osteoblastogenesis, triggering us to take miRNAs special for osteogenic differentiation into consideration.

## 3. *In Vitro* Profiling Analysis of Osteogenesis-Related MiRNAs

As mentioned above, osteogenesis is a delicately regulated process requiring proper osteoblast activities altered by hundreds of miRNAs. For the moment, miRNA profiling methods include varied quantitative RT-PCR (qPCR) analysis, such as qPCR with locked nucleic acid primers (Exiqon), high throughput sequencing of small RNA libraries, and microarray analysis [26]. MiRNA expression profiles by microarray analysis can provide us diverse miRNAs whose levels change during osteoblast differentiation, which is a commonly used technique for genome-wide miRNA expression analysis. To identify and understand differentially expressed miRNAs related to the osteoblast differentiation program, miRNA array profiling has been performed by many research groups in various cell lines during induced osteogenesis. In bone morphogenetic protein (BMP)-2 induced osteoblast differentiation of mouse C2C12 mesenchymal progenitor cells, 36% of the miRNAs are down-regulated, while only 4% of them are up-regulated [27]. In concert with this study, 22 of the 25 miRNAs were observed to be reduced in BMP-2-treated C2C12 cells [28]. In addition, 31 of the 51 altered miRNAs are decreased in MC3T3-E1 murine osteoblast-like cells and 6 of the 20 altered miRNAs are reduced in MLO-A5 murine preosteocyte-like cells [19]. Also, 58 miRNAs are up-regulated and 10 are down-regulated during the mineralization stage of the murine calvaria-derived preosteoblasts (MC3T3 cells) [29]. In human unrestricted somatic stem cells (hUSSCs), Trompeter *et al.* depicted that 124 miRNAs are up-regulated in SA5/73 cell line while 196 miRNAs are up-regulated in SA8/25 cell line during osteogenic differentiation, among which 30 are increased in both USSC lines. They also showed that miR-10a, miR-22, miR-26a/b, miR-29b, miR-30b/c, miR-152, miR-345, and miR-532-5p are the most prominently expressed ones [30]. A study on the differentiation of hBMSCs towards adipogenic, osteogenic, and chondrogenic lineages displayed the up-regulation of miR-196a, miR-378-star, miR-486-5p, and miR-664-star with the down-regulation of miR-10a, miR-708, and miR-3197 from old subjects (ranging from 65 to 80 years old) compared with young subjects (ranging from 17 to 30 years old) [31]. Another research by Baglìo *et al.* demonstrated that 17 miRNAs are up-regulated and 12 are down-regulated in differentiated hBMSCs. Interestingly, during the mineralization stage, miR-183, miR-376a, miR-520g-520h, miR-607, and miR-611 were observed to be up-regulated, whereas miR-302a, miR-508, miR-520a, miR-520b-520c, and miR-520f-520c were observed to be down-regulated [32]. Moreover, levels of 33 miRNAs are considerably modulated between differentiated and non-differentiated hBMSCs, among which miR-26a, miR-26b, miR-30c, miR-101, and miR-143 are up-regulated and miR-138 and miR-222 are down-regulated during osteoblast differentiation [17]. Furthermore, let-7, miR-20, miR-29, miR-34a, miR-34c, miR-125, miR-138, miR-148, and miR-199 were detected to be augmented during osteoblast differentiation of hBMSCs by Chen and his group [33].

Although a sea of miRNAs have been verified to either ascend or descend during bone formation, which we have discussed in the previous part, only few of them are significantly altered and have potential targets involved in skeletal development proved by subsequent studies, meaning that they are more valuable for us to further investigate. In other words, miRNAs changed most obviously during osteogenesis are candidates, but those that are really relevant to genes involved in osteoblast differentiation according to the biological database or literature should be paid attention to. Although some miRNAs are increased greatly during bone development, they are particularly engaged in the regulation of other important genes such as some housekeeping genes that are not specific to skeletons. Here, we will deliberate the biological dynamic characteristics of some osteo-related miRNAs (Table 1).

MiR-335-5p was reported to be up-regulated during osteoblastogenesis of MC3T3-E1 and MLO-A5 cells. It also increases in C3H10T1/2 murine mesenchymal stem cells at early time points but decreases later [19]. Li and his group demonstrated that miR-29b reaches its peak in the period of mineral deposition during osteoblastic differentiation of MC3T3 cells as well as primary rat calvaria osteoblasts [29]. Bhushan and his colleagues induced osteoblastic differentiation of C2C12 and MC3T3 cells using BMP-2 and BMP-6, respectively, and depicted the up-regulation of miR-181a in both cell lines. Moreover, they confirmed that the miR-181 family members including miR-181b and miR-181c increase in tibia and calvarial tissues *in vivo* as well as during *in vitro* differentiation [34]. The expressions of miR-2861 and miR-3960 are up-regulated gradually during BMP-2-induced osteoblastogenesis of the mouse bone-marrow-derived stromal cell line ST2. Also, miR-2861 that may involve in human osteoporosis can enhance bone formation and increase bone mass in mice [20,21]. In the same cell line, miR-210 was shown to increase in a BMP-4 dose-dependent manner [35].

In the osteogenic differentiation of hADSCs, miR-218 expression level was observed to be augmented [36]. Similar to miR-218, miR-196a expression level increases from day 6 after the induction of osteogenic differentiation of hADSCs [37]. Furthermore, the experimental data obtained by Wang *et al*. on miR-346 elucidated that it ascends gradually during osteogenic differentiation of hBMSCs [38]. MiR-34a is also up-regulated during bone formation of hBMSCs, while miR-34b reveals modest regulation [33]. Intriguingly, a study directed by Vimalraj *et al.* examined pre-miR-15 and showed that the expression of this pre-miRNA increases during skeletal formation of hBMSCs and it is highly enriched in rat osteoprogenitor cells as well [39]. Actually, the fact that the expression of roughly 60% miRNAs detected in primary effusion lymphoma relevant to the levels of their precursors has been validated [40]. Therefore, it arouses the consideration of not only examining mature miRNAs, but also investigating their precursors, which may help decipher the essence of the biological functions of these small noncoding RNAs.

Apart from being up-regulated, some miRNAs are decreased during skeletogenesis. MiR-93 is reduced during osteoblast differentiation and mineralization of primary mouse osteoblasts from calvaria [41]. The expression of miR-338-3p is significantly down-regulated during osteoblast differentiation of mouse BMSCs [42]. MiR-378 level increases considerably at early stages of osteogenesis in MC3T3-E1 cells and declines rapidly during cell differentiation [43]. In BMP-2-induced osteogenesis of mouse C3H10T1/2 cells, miR-433 level was observed to be down-regulated [44].

**Table 1:** Dynamic changes of miRNAs during osteogenesis.

MiRNA	Variation Trend	Cell Line	Reference
pre-miR-15	+	hBMSC	[39]
miR-29b	+	MC3T3 primary rat osteoblasts	[29]
miR-34a	+	hBMSC	[33]
miR-93	−	primary mouse osteoblast	[41]
miR-138	−	MC3T3 hBMSC	[17]
miR-140-5-p	−	hBMSC hADCS hUCSC	[22]
miR-181a	+	C2C12 MC3T3	[34]
miR-196a	+	hADSC	[37]
miR-210	+	ST2	[35]
miR-218	+	hADSC	[36]
miR-335-5p	+	MC3T3 MLO-A5 C3H10T1/2	[19]
miR-338-3p	−	mouse BMSC	[42]
miR-346	+	hBMSC	[38]
miR-433	−	C3H10T1/2	[44]
miR-2861	+	ST2	[20]
miR-3960	+	ST2	[21]

+: increased during osteogenesis; −: decreased during osteogenesis.

In hBMSCs and mouse calvarial MC3T3-E1 cells, miR-138 is down-regulated during osteoblast differentiation [17]. MiR-140-5-p decreases during the early phase of osteogenic differentiation of hMSCs from different tissue sources, which is inversely correlated with the expression level of BMP-2 [22]. Taken together, there are various miRNAs participating in the development of osteoblasts and they exhibit varied dynamic patterns during osteogenesis, which is a fine-tuned process. However, those that are up-regulated may exert positive functions, while those that are down-regulated possibly exert negative functions on skeletal formation.

## 4. MiRNAs and Bone Development

### 4.1. MiRNAs and Osteo-Related Transcription Factors in Bone Formation 

MiRNAs target mRNAs via complementary base-pairing to multiple sites in the 3'-untranslated regions, and the 5'-end of miRNA nucleotides of 2 to 8 are called seed region [45]. Generally speaking, miRNAs exert their biological functions either by directly suppressing the translation or by deadenylation and subsequent degradation of various mRNA targets [1,45]. Upon regulating the proliferation and differentiation of osteoblasts, they precisely control the activation or suppression of lots of osteo-related genes (Table 2). Recently, an increasing number of transcription factors have been confirmed to make vital contributions to the precise control of skeletal development, among which Runx2 and its downstream molecule Osterix are the most paramount osteoblast-specific transcription factors that activate a repertoire of genes during the differentiation of preosteoblasts into mature osteoblasts and osteocytes.

Runx2 (runt-related transcription factor 2, also known as core binding factor alpha 1 (Cbfa1), osteoblast specific transcription factor 2 (Osf2) and acute myeloid leukemia 3 protein (AML3)) is a bone restricted transcription factor essential for osteoblast differentiation and bone formation, which was validated by many researches decades ago, and mutations in Runx2 are found in 65%–80% of individuals with cleidocranial dysplasia [46,47]. Runx2-deficient mice were found to show a complete lack of skeletogenesis owing to the maturational arrest of osteoblasts [48]. Runx2 synergistically increases osteogenic gene expression, including osteocalcin (OCN), osteopontin (OPN), type I collagen (ColI), bone sialoprotein (BSP), and alkaline phosphatase (ALP). It also promotes biological mineral deposition in primary dermal fibroblasts [49]. MiRNAs can interact with Runx2 through direct targeting its gene or they may affect other genes that enhance or inhibit Runx2 expression level. MiR-133 targets Runx2 mRNA, thereby inhibiting BMP-2-induced osteoblast differentiation of mouse C2C12 cells [28]. In mouse BMSCs, miR-338-3p that directly affect Runx2 and fibroblast growth factor receptor 2 (Fgfr2) serves as a negative regulator of osteogenic differentiation and may also contribute to osteoporosis, which was shown up-regulated in ovariectomized (OVX) mice compared with sham mice [42]. Jeong’ group reported that ERRγ, namely estrogen receptor-related receptor γ, negatively regulates osteoblast differentiation by inhibiting Runx2 transactivity in pre-osteoblast MC3T3-E1 or primary calvarial cells. In their follow-up experiment, ERRγ was shown to induce miR-433 directly modifying Runx2 via a post-transcriptional manner in murine C3H10T1/2 cells, thus repressing BMP-2-induced bone formation [44]. Furthermore, Zhang *et al.* reported that miR-23a, miR-30c, miR-34c, miR-133a, miR-135a, miR-137, miR-204, miR-205, miR-217, and miR-338 can regulate *Runx2* gene, which significantly blocked MC3T3 osteoblast differentiation [50,51].

It was well documented that Smad specific E3 ubiquitin protein ligase 1 (Smurf1) degrading Runx2 through proteasomal pathway is identified as a target gene for miR-15b which can help indirectly boost Runx2 level by reducing Smurf1 in hBMSCs [39]. A novel study which investigated the mechanical stimulation for triggering osteoblast differentiation shed light on mechano-sensitive miRNAs in cyclic mechanical stretch (CMS)-induced osteoblast differentiation of human preosteoblast cell line hFOB1.19 for the first time. And miR-103a was detected a mechano-sensitive miRNA that inhibits osteoblast activity and extracellular matrix mineralization via targeting Runx2 at the post-transcriptional level [52]. MiR-204 and miR-211 play a negative role in osteogenesis by reducing Runx2 protein level but promote adipogenesis of hMSCs as well as ST2 cells [18]. In a recent model of osteogenic differentiation of human dental stem cells (hDSCs), the expression level of Runx2 was presented to be down-regulated by miR-218 [53]. Also, miR-106a, miR-125b, miR-148a, miR-335, miR-355, miR-424, and miR-3077 were confirmed to correlates with Runx2 [45,54]. While several recent papers brought insight into the mechanisms that how miRNAs control Runx2 function, more studies are still needed to unravel the exact network among miRNAs, Runx2 and osteoblast differentiation [10,45,54].

Osterix (Osx, Sp7), another vital transcription factor containing zinc-finger structure, belongs to the Sp subgroup of the Kruppel-like family. It was initially identified by subtractive screening of BMP-2 induced genes in osteoprogenitor cells compared to untreated controls and validated to be essential for osteogenesis and embryonic skeletal development owing to its enhancing the expression of several osteogenic factors via binding to the specific GC-rich sequences. Osx-null mice die at birth due to their lack of mineralized skeletons. The mouse Osx homolog is a 428 amino acid polypeptide with a molecular mass of ~45 kDa [55,56,57]. Research on miR-93 showed that, in cultured primary mouse osteoblasts, mineralization is inhibited by overexpression of miR-93, which targets *Osx* gene [41]. Li *et al.* reported that miR-143 acting as a suppressor in several tumors such as non-small cell lung cancer, pancreatic cancer and breast cancer negatively regulates the osteogenic differentiation of MC3T3-E1 cells by reducing Osx expression [58,59,60,61]. MiR-145 was displayed to reduce Osx expression, thus repressing osteogenic differentiation of C2C12 and MC3T3-E1 cells [62]. Another research on BMP-2-induced osteogenic differentiation of C2C12 cells revealed miR-214 as a suppressor of Osx [63].

MiR-31 was reported to degrade Osx mRNA expression, leading to osteogenic inhibition in hBMSCs [32]. Decreased miR-125b level was detected during osteogenic transdifferentiation of human coronary artery smooth muscle cells (hCASMCs) and inhibition of endogenous miR-125b targeting Osx can promote osteogenesis [64]. In accordance with this study, an up-regulated expression level of miR-125b was observed in osteoporotic hBMSCs and it suppresses the proliferation and osteogenic differentiation of hBMSCs through down-regulating Osx expression level [65]. MiR-637 also negatively modulates osteoblast differentiation and enhancing adipocyte differentiation of hBMSCs through direct suppression of Osx expression [66]. Besides, miR-31, miR-142 and miR-138 were exhibited to repress Osx expression, whereas miR-322 was shown to up-regulate Osx level [54,67].

In addition to Runx2 and Osx, there are many other important factors involved in skeletal metabolism, which are also precisely tuned by miRNAs. As a matter of fact, most of them are either upstream or downstream factors, cofactors, or binding partners of Runx2, Osx and other crucial transcription factors. They also participate in diverse signaling pathways such as BMP/TGF-β and Wnt/β-catenin. MiR-125b was displayed to repress BMP-2-induced osteoplastic differentiation of mouse C3H10T1/2 cells by reducing the mRNA and protein levels of core binding factor beta (Cbfβ), a key transcription factor for osteogenesis, which was marked by decreased expression of ALP, OCN and OPN [68]. Itoh *et al.* showed that both miR-141 and miR-200a remarkably modulate the BMP-2-induced bone formation in mouse MC3T3 cells through the translational repression of distal-less homeobox 5 (Dlx5), a bone-generating transcription factor expressed in pre-osteoblast differentiation [69]. Activating transcription factor 4 (ATF4) protein improves osteoblast-specific gene expression, amino acid uptake by the cells and synthesis of type I collagen, thus enhancing osteoblast activity. It is targeted by miR-214 that inhibits bone formation in mouse preosteoblast MC3T3-E1 cells. This was further verified in two mouse models [70].

Li’s group compared miRNA expression profiles of hBMSCs derived from young and old individuals and found that miR-10a is the most significantly altered with aging. Their further results revealed that miR-10a can enhance hBMSC differentiation towards different cell lineages including osteoblasts via repressing Kruppel-like factor 4 (KLF4) [31], a conserved zinc finger transcription factor essential for somatic cell reprogramming [71]. In a recent study by our group, we illustrated that miR-31 which modulates special AT-rich sequence-binding protein 2 (SATB2) expression by interfering with its mRNA translation performs as a negative regulator of the osteogenesis of human hADSCs. SATB2 plays a critical role in regulating osteogenesis, it has been reported to not only bind individually to the promoter of osteogenic specific genes, but also synergistically enhance the regulatory role of Runx2 [72,73]. Focal adhesion kinase (FAK) translation is repressed by miR-138. As a consequence, its phosphorylation and downstream target extracellular signal regulated kinase 1/2 (ERK1/2) are decreased, resulting in reduced phosphorylation of Runx2 and expression of Osx during osteogenesis of hBMSC [17]. An up-regulation of jumonji domain containing 3 (JMJD3), a histone demethylase targeted by miR-146a, and a down-regulation of miR-146a were observed in the osteogenic differentiation of hUCSCs [74]. Elevated miR-196a can decrease the mRNA and protein expressions of homeobox C8 (HOXC8), a transcriptional repressor interacting with Smad-1, causing impaired proliferation but enhanced osteogenic differentiation of hADSCs [37].

### 4.2. Other Target Molecules of MiRNAs in Skeletogenesis

Here, we use “other” to describe these molecules, but do not mean that they are less vital. The authors just desire to distinguish them from the classic transcription components contributing to bone development. Yet, these so-called “non-classic” targets are numerous, complex, proteins performing a variety of biological functions that facilitate bone development (Table 2). MiR-29a and miR-29c induced by Wnt signaling during bone formation positively regulates osteoblast differentiation of mouse MC3T3-E1 cells by controlling the expression of osteonectin that contributes to bone mass in mouse models and is associated with bone mass in some osteoporosis patients [75]. Rgs4 and Gata6 are direct targets suppressed by miR-181a during osteoblastic differentiation in MC3T3 cells, whereas Gadd45b still needs to be further studied [34]. Inose and his colleagues proved that, in C2C12 cells, miR-206, which is previously viewed as a muscle-specific miRNA and only expressed in vertebrates, decreases during osteoblast differentiation while overexpression of it inhibits bone formation partially by targeting connexin 43. They conjectured that miR-206 may work to keep osteoblast immature [27]. Kahai *et al.* described that over-expression of miR-378 inhibits osteoblast differentiation of MC3T3-E1 cells. Moreover, they established stable cell line where they co-transfected nephronectin (Npnt)-3'-UTR, an extracellular matrix protein that can induce osteoblastic differentiation, with miR-378. It is worth noting that, in this model, miR-378 reversed its function with Npnt-3'-UTR, a potential target for miR-378. It was suggested that a space hindrance preventing direct contact with other miRNAs may be created by miR-378 via binding to the 3'-UTR of nephronectin mRNA, which brings about incremental synthesis of nephronectin protein level rather than repression. As a result, the true target of miR-378, namely polypeptide *N*-acetylgalactosaminyltransferase 7 (GalNT7) essential for nephronectin glycosylation, can be restored, which is congruent with the elevated nephronectin protein synthesis and glycosylation [43]. Recently, research by Hupkes *et al.* demonstrated that miR-378 promotes BMP-2-induced osteogenic differentiation of C2C12 cells. Yet, they were unable to identify the target genes [76].

Chen *et al*. observed that miR-34a correlates with the expression of *CyclinD1*, *CDK4*, *CDK6* and *E2F3* genes governing cell cycle in hBMSCs, which is consonant with previous researches [77,78,79]. Furthermore, they indicated that miR-34a arrests hBMSC cell cycle at G1 and G2 phases and a novel target, Jagged1 (JAG1), is detected. It plays a vital role in human bone metabolism, as is shown in Alagille syndrome (AS) [80,81]. MiR-34a is able to suppress JAG1 level both transcriptionally and post-transcriptionally [33]. Because of space constraints, we cannot delineate all of the miRNAs reported in the contemporary era. Gratifyingly, some elegant reviews have been issued recently, elaborating the functional miRNAs and their potential targets in the skeleton (For further information, see [10,45,54,67]).

### 4.3. The Interplay between MiRNAs and Signaling Pathways in Bone Homeostasis

Skeletal development and bone formation require coordinated activities of multiple signaling pathways, and understanding the diversity of these signals remains as a challenge. Different signaling components assemble sophisticated networks where osteoblast lineage-specific master genes and their cellular substrates exhibit intimate reciprocity [82]. In addition, osteoblast proliferation and differentiation are carefully orchestrated by miRNAs as well (Table 2 and Table 3).

BMPs are multi-functional growth factors that belong to the transforming growth factor beta (TGF-β) superfamily, which are potent osteogenic agents that stimulate maturation of mesenchymal osteoprogenitor cells to osteoblast upon the well-known Smad-dependent BMP signaling pathway [83,84]. BMP signals through type I and type II serine/threonine kinase cell membrane receptors. Specific receptor-regulated Smads (R-Smads) serve as substrates for the BMP and TGF-β/activin/Nodal receptors. In the canonical pathways, Smad-1, -5, and -8 are activated, while Smad-4 serves as a common partner to form complexes with them, providing the DNA binding property. The complexes then translocate to the nucleus to activate specific gene transcriptions [85,86,87].

The research of Li *et al*. indicated that, in MC3T3 cells, miR-29b represses TGF-β3 as well as activin A receptor type II A (AcvR2a), another member of the TGF-β superfamily, which helps to maintain the levels of some vital transcription factors required for bone formation like Runx2. MiR-135a, down-regulated in BMP-2-induced osteoblast differentiation of mouse C2C12 cells, interacts with 3'-UTR sequence of Smad-5 mRNA and negatively regulate bone development [28]. In BMP-2-stimulated MC3T3 cells, miR-181a was confirmed to promote osteogenesis by impeding transforming growth factor beta-induced (TgfbI), an extracellular matrix protein induced by TGF-β, and TGF-β type I receptor (TβR-I) protein level [34]. Furthermore, the decreasing of Smad-1 mRNA expression level by miR-199a treatment can hinder BMP-2 signaling pathway in mouse C3H10T1/2 cells [88]. Mizuno *et al.* investigated the effect of miR-210 on osteogenesis in BMP-4-induced mouse ST2 mesenchymal stem cells and found that it promotes osteoblastic differentiation through inhibition of the activin A receptor type I B (AcvR1b, also known as activin receptor-like kinase 4 (Alk4)) gene, thereby blocking signals from TGF-β/activin pathway and further inhibiting the phosphorylation of Smad-2 and Smad-3 [35]. MiR-542-3p directly targets BMP-7 and inhibits its protein translation, thus down-regulating the phosphorylation of protein kinase B (Akt) and survivin that help activate caspase-3, which leads to osteoblast apoptosis. In addition, miR-542-3p can also impede Smad-dependent BMP signaling pathway thanks to the reduced BMP-7 level, resulting in impaired differentiation of mouse calvaria-derived primary osteoblasts [89].

As a suppressor, miR-26a participates in the osteo differentiation of hADSCs by diminishing the availability of the active Smad-1, leading to more HOXC8 binding to DNA and less OPN expression [90]. MiR-125b targets the 3'-UTR of Smad-4 mRNA, resulting in the inhibition of osteogenesis in hBMSCs through interfering with the formation of Smad-1, -5, -8, and -4 complexes [91]. MiR-140-5p inhibits osteogenic differentiation of different hMSCs via reducing both BMP-2 mRNA and protein levels and it further down-regulates the protein levels of Smad-5, phospho-Smad-1/-5 and bone morphogenetic protein receptor type II (BMPR2) in the downstream of BMP signaling pathway [22]. Cheung *et al*. demonstrated that miR-146a promotes skeletogenesis via attenuation of Smad-2 and Smad-3 protein translation in human fetal femur derived skeletal stem cells (hSSCs) [92]. This is in direct contrast with the results of Huszar *et al.* in hUCSCs [74], and indicates that the effect of miR-146a may depend on osteogenic model system used and/or the signaling pathways involved in inducing differentiation. MiR-654-5p can suppress skeletogenesis of hBMSCs by repressing BMP-2 mRNA [93]. Moreover, miR-15b targeting Smurf1, miR-17 targeting Smurf1, miR-30 targeting Smad-1, miR-31 targeting BMPR1, miR-133 targeting Smad-5, miR-155 targeting Smad-1 and -5, and miR-208 targeting Protein C-ets-1 (Ets1) were validated to be involved in BMP/TGF-β signaling pathway as well [45,54,67,94].

Wnt signaling is involved in diverse processes including embryonic development, maintenance of tissue homeostasis, and cancer pathogenesis. It also affects bone development, especially the differentiation of osteoblasts [95,96]. Canonical Wnt signaling occurs through the binding of extracellular Wnt ligands to the seven-pass transmembrane Frizzled receptor and the co-receptor LRP5/6 complex containing intracellular proteins of the disheveled (DSH) family, which inactivate a multiprotein destruction complex of β-catenin with axis inhibition protein (axin), glycogen synthase kinase 3 (GSK3), and adenomatosis polyposis coli (APC) protein. As a consequence, β-catenin will be stabilized [96,97,98].

The mRNA and protein levels of histone deacetylase 4 (HDAC4) can be repressed by miR-29a. Hence, more acetylated β-catenin will accumulate and osteoblast differentiation capacity of murine MC3T3-E1 cells is sustained [99]. Similarly, miR-29b targets HDAC4 and catenin beta interacting protein 1 (CTNNBIP1, also known as isotope coded affinity tag (ICAT)), improving β-catenin-mediated transcription [29]. Collectively, the members of the miR-29 family are key regulators for the development and maintenance of the osteoblast phenotype. MiR-335-5p was depicted to activate Wnt/β-catenin signaling pathway with increased GSK-3β phosphorylation and enhanced β-catenin transcriptional activity, and promote osteogenic differentiation of murine C3H10T1/2 cells by down-regulating dickkopf-related protein 1 (DKK1) [19].

Wnt signaling is activated by miR-27 upon targeting APC and inhibiting its gene expression, therefore, promoting osteoblastic differentiation of hFOB1.19 cells [100]. Some miRNAs stimulate the Wnt signaling pathway via impeding its antagonists during the process of osteogenesis: secreted frizzled-related protein1 (SFRP1) by miR-27a in hFOB1.19 cells, DKK1, Kremen2, and SFRP2 by miR-29a in hFOB1.19 cells, DKK2 and SFRP2 by miR-218 in hADSCs, and Sclerostin (SOST), DKK2, and SFRP2 by miR-218 in MC3T3-E1 cells [36,101,102,103]. MiR-346 was proved to stimulate osteogenesis in hBMSCs by down-regulating GSK-3β protein expression at a post-transcription level and activating Wnt/β-catenin signaling pathway through increasing total β-catenin quantity [38]. Tissue inhibitor of metalloproteinases 1 (TIMP-1), a glycoprotein, is a negative regulator of the growth and osteogenic differentiation of hBMSCs. And knockdown of TIMP-1 accelerates osteogenesis through enhancing the activity of β-catenin and up-regulating let-7f that targets axin2, an antagonist of β-catenin stability [104].

Apart from BMP/ TGF-β and Wnt transduction, other signaling pathways, such as MAPK signaling, Notch signaling, RANK-OPG-RANKL signaling, Hedgehog Signaling, NELL-1 signaling, IGF signaling, and oxidative stress signaling pathways, all govern MSC fate towards osteoblastogenic differentiation or other cell types [98,105]. Take miR-34c as an example. In osteoblasts, it decreases multiple components of the Notch signaling pathway, including Notch1, Notch2 and Jag1, *in vivo* [106]. Another example is that miR-29b down-regulates dual specificity phosphatase 2 (DUSP2) that inactivates and anchors extracellular regulated MAP kinase (ERK) within the nucleus, influencing the MAPK signaling during osteogenic differentiation [29]. Yet, more work is urgently needed to elaborate miRNAs and their diverse regulatory functions in those different pathways. Collectively, understanding the complicated molecular networks between miRNAs and varied signaling pathways involved in bone formation and metabolism will have significant implications in the therapeutic approaches for artificial skeletal regeneration.

### 4.4. Coordinate Regulation by Feedback Loops of MiRNAs in Osteoblasts

Physiological feedback loops are quite common in almost all the facets of biologic processes. Since several previous studies unraveled a few regulatory circuits for induction of osteogenesis and regulation of the progression of differentiation, we are required to pour our attention to how these loops properly interact with each other. An exquisitely designed study on the miR cluster 23a~27a~24-2 presents us with a general idea regarding these networks. Each miRNA in the cluster suppresses osteoblast differentiation of MC3T3 cells upon targeting SATB2, which facilitates ostogenesis with Runx2, while Runx2 negatively regulates the level of the cluster via a Runx regulatory element in the promoter of this cluster, which leads to elevated Runx2 and SATB2 expression. Furthermore, miR-23a can repress Runx2 in the terminally differentiated osteocyte [107]. As previously mentioned, miR-29a targets inhibitors of Wnt signaling in hFOB1.19 cells as well as HDAC4 that accelerates the degradation of β-catenin in MC3T3-E1 cells, while in turn this pathway can induce miR-29a transcription [99,102]. Furthermore, Kapinas *et al*. disclosed that, in the later stage of osteogenesis, osteonectin synthesis is attenuate by increased miR-29a in MC3T3-E1 cells [75]. This is in line with the fact that extracellular matrix proteins are down-regulated with more differentiated osteoblasts and enhanced Wnt signaling pathway [108]. It was proved by our group that miR-31 diminishes SATB2 protein expression and inhibits osteogenic differentiation of rat BMSCs. Yet, it does not affect Runx2 level, whereas Runx2 directly represses miR-31 expression [109]. A study conducted by Yang’s group demonstrated that overexpression of miR-93 attenuates osteoblast mineralization by repressing the protein levels of osterix. In turn, osterix bound to the promoter of miR-93 transinactivates miR-93. Their study established a favorable regulatory feedback loop between miRNA and its target [41]. An earlier study by the same group developed another feedback loop between runx2 and miR-3960/miR-2861 found clustered at the same loci in the mouse stromal cell line ST2. When runx2 is activated during osteoblast differentiation, it targets the promoter and transactivates miR-3960/miR-2861 cluster, which preserves the expressions of runx2 mRNA and protein via down-regulation of homeobox A2 (HOXA2) and HDAC5 levels respectively [21]. From the study on homozygous Osx^−/−^ mice, Chen and his colleagues demonstrated that Osx can decrease a set of miRNAs including miR-133a, miR-204 and miR-211 and increase another group of miRNAs like miR-141 and miR-200a. Intriguingly, miR-133a, miR-204 and miR-211 were verified to down-regulate Runx2 level, while miR-141 and miR-200a were confirmed to target Dlx5, which is essential for osteoblastogenesis. As a downstream molecule of Runx2, their research delineated the possible ability of Osx to coordinately modulate Runx2 level and corresponding miRNAs involved in skeletal formation [110]. As stated above, the Wnt signaling pathway is stimulated by miR-218 through weakening the expressions of SOST, DKK2 and SFRP2 in MC3T3 osteoprogenitors. Accordingly, the level of miR-218 is increased in response to improved Wnt signaling that promotes osteoblast differentiation, thereby creating a positive feedback loop [103]. In hADSCs, miR-218 stimulates osteogenesis by impeding Wnt antagonists, DKK2 and SFRP2 at mRNA and protein levels, thus activating Wnt/β-catenin signaling pathway, which in turn enhances expression of endogenous miR-218. It is suggested that miR-218 acts as a signaling amplifier for positively regulating the osteogenic differentiation of hADSCs [36]. This current result aligns with the outcome depicted in the study on MC3T3-E1 cells.

In aggregate, both multi-target effects and regulatory networks are situated at the heart of maintaining a normal micro-environment for skeletons. Comprehending these circuits will equip us with additional insight into how miRNAs interact with bone tissue differentiation pathways, which allows for seeking novel ways of bone repair.

## 5. MiRNAs and Bone Regeneration

**Figure 1:**
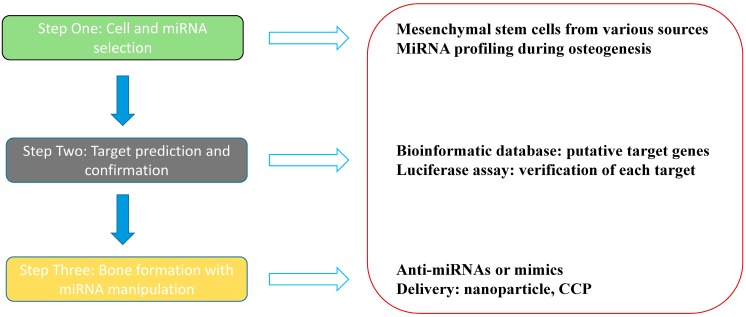
MiRNA-based skeletal regeneration.

Skeletal development includes the fine coordination of multiple biological events characterized by an increased potential for growth, regeneration and remodeling throughout life. Bone mass and density loss leads to weakened bone strength, which accounts for an increase in the propensity of bone fracture. Therefore, the repair of bone defects is a must at present, which remains a major clinical orthopedic challenge (Figure 1).

In virtue of osteoblasts originating from the bone marrow, the first of all our priority is to search for the precursors suitable for regeneration: the mesenchymal stem cells, which contribute to the regeneration of mesenchymal tissues such as bone, cartilage, muscle, ligament, tendon, adipose, and stroma [111]. After transplantation of MSCs, skeletal myoblasts, cardiac myoblasts, endothelium, hepatic and biliary duct epithelium, lung, gut and skin epithelia, and neuroectodermal cells of donor origin have been detected [112]. The formation of bone tissue begins with the differentiation of bone marrow stromal stem cells into immature osteoprogenitors that further turn into the pre-osteoblasts. Then, these precursor cells become mature osteoblasts. MiRNAs are detected in each of the delicate step, playing either a positive or negative role in osteogenesis [10,54,67,94]. Gene and miRNA expression analysis enable us to reveal specific miRNAs modulating MSCs towards different cell lineages such as miR-96, miR-124, and miR-199a expressed differently in osteogenic, adipogenic, and chondrogenic induction [113]. The following work is to determine various miRNA levels change between undifferentiated and osteo-differentiated MSCs, for e.g., miR-30c, miR-15b, and miR-130b overexpressed in osteoblasts [114].

The second task for bone remodeling is to identify and study the putative targets of miRNAs. Bioinformatic target gene predictions with web-based algorithms such as TargetScan, PicTar-4, PicTar-5, miRanda, and DIANA microT [26,28,30] can provide numbers of potential targets of miRNAs, which should be further corroborated using luciferase assay or other techniques. On the one hand, classic osteo transcription components such as SATB2 suppressed by miR-34b as well as miR-34c in primary mouse osteoblasts is still being detected contemporarily [115]. On the other hand, numerous novel or underreported targets such as PPARγ repressed by miR-20a and miR-548d-5p, Bambi and Crim1 both silenced by miR-20a in hBMSCs are emerging rapidly [116,117]. Besides the 3'-UTR regions, it is intriguing that miRNAs may exert biologic functions on their natural target genes in the amino acid coding sequence (CDS), which assists in scientists’ apprehending the diverse patterns of these small molecules’ activities. Liu *et al*. found that both CDS and 3'-UTR targets are efficiently repressed by miR-15a/miR-16 and miR-92a [118]. A well-designed study conducted by Tay *et al*. illustrated that miR-134 targeting sex-determining region Y (SRY)-related HMG box 2 (Sox2), miR-296 targeting Nanog homeobox (Nanog), and miR-470 targeting both Nanog and octamer-binding transcription factor 4 (Oct4, also known as POU domain class 5 transcription factor 1 (Pou5f1)), which are up-regulated on retinoic-acid-induced differentiation of mouse embryonic stem cells, acted on the coding regions, resulted in transcriptional and morphological changes characteristic of these differentiating cells [119]. These above studies allow us to wonder whether there also exists such peculiar way of miRNAs’ action on osteogenesis. Apart from their post-transcriptional control on mRNAs, miR-2861 has already been proved to bind to the CDS of HDAC5 mRNA, while miR-3960 has been confirmed to interact with the CDS of HOXA2 mRNA [20,21]. Moreover, the suppressive effect of miR-93 on the CDS of osterix mRNA was also verified by luciferase reporter assay [41]. All of these current achievements render us capable of manipulating bone repair and regeneration through modulating various osteo-related miRNA expression levels.

The last but not least assignment should be focused on how to control miRNA activities in an anabolic bias way. In skeletogenesis, the method lies at using anti-miRNAs to impede those targeting promoter protein coding genes, and mimics to enhance those targeting inhibitor protein coding genes [45]. For inhibition of miRNA function, antisense miRNA oligonucleotides and microRNA sponges are widely applied. A novel class of chemically modified, cholesterol-conjugated single-stranded RNA analogues complementary to miRNAs has been designed, and these antisense oligonucleotides (ASOs) are termed “antagomirs” [26]. In 2007, Ebert and his colleagues developed another kind of microRNA inhibitors that can be expressed in mammalian cells and they named them “microRNA sponges”. These competitive inhibitors are transcripts expressed from strong promoters, containing multiple, tandem binding sites to a microRNA of interest [120]. Overexpression of a specific miRNA is often achieved by viral-based methods, among which adeno or adeno-associated viral vectors as well as lentiviral and retroviral vectors are the most commonly used ones [121]. Likewise, this aim can also be achieved via transient transfection of double stranded miRNAs, which doesn’t enable long-term expression as viral vectors [21]. Inspiringly, new researches shed light on novel vehicles able to deliver miRNAs into the cells. Qureshi *et al.* used a silver nanoparticle complex for photoactivated miR-148b mimic delivery, resulting in osteogenic differentiation of hADSCs [122]. Another study illustrated the possibility of a cell-penetrating peptide (CPP) rich in arginine, also called the low molecular weight protamine (LMWP), mediating intracellular delivery of miR-29b for osteogenesis of hMSCs [123]. Consequently, the existing methods are just a tip of the iceberg for miRNA/anti-miR delivery technology. More issues remain to be settled for exploring better materials with lower levels of toxicity and less off-target effects.

Either way, the ultimate goal of miRNA-based skeletal regeneration lies in whether and how they can be used to regenerate bone *in vivo*. Wei and his colleagues carried on a valuable research into miR-34 family with miR-34s-deficient mice and miR-34c transgenic mice and well illuminated the functions of miR-34s in osteoblasts [115]. Analogically, miR-338-3p was shown to be up-regulated in a murine model of ovariectomy induced osteoporosis, which was further proved to be a negative regulator of osteogenesis [42]. While these groups simply elaborated the vital role of some miRNAs in skeletogenesis, they did not go further into *in vivo* bone repair or regeneration study. Nonetheless, some progress has been made in this facet. Eskildsen *et al.* showed by hematoxylin-eosin (H.E.) staining that hydroxyapatite/tricalcium phosphate (HA/TCP) ceramic powder with anti-miR-138 transfected hBMSCs enhanced bone formation in nonobese diabetic/severely compromised immunodeficient (NOD/SCID) mice [17], which is an admirable study of *in vivo* heterotopic bone formation. Recently, another preclinical murine model of heterotopic osteogenesis was conducted by Chen *et al.*, in which either pre-miR-34a or anti-miR-34a was transfected into hMSCs that were loaded on HA/TCP scaffold implanted subcutaneously in NOD/SCID mice, and newly formed bone was detected by H.E. staining with anti-miR-34a transfected cells [33]. While bone volume was quantified with quantitative histological method in these two researches, it would be more persuasive to combine the micro-computed tomography (micro-CT) examination and *in situ* bone defect study rather than ectopic bone formation.

It is generally recognized that the assessments of bone density, microarchitecture and strength are significant in skeletal formation/regeneration research field [124]. In recent years, high-resolution micro-CT has become feasible for *in vivo* evaluation of bone quality, providing a high possible imaging spatial resolution [125]. Therefore, a number of research groups concentrate on the application of this advanced technology to appraise bone quality with miRNA treatment. For instance, in work by Inose *et al.* significantly decreased bone mass was confirmed by both micro-CT and histological analysis in miR-206 transgenic mice [27]. Recently, some *in vivo* bone repair studies have been released. In a hind limb unloading mouse model, micro-CT revealed that bone loss was partly rescued by anti-miR-103a treatment [52]. What is more, silencing of miR-542-3p led to an increased bone mass gain by micro-CT examination in OVX mice [89]. An earlier research by Wang *et al.* focusing on miR-214 displayed notably improved bone regeneration with anti-miR-214 treatment verified by both murine models [70].

Finally, we expect to deepen readers’ comprehension of miRNA-mediated bone repair through delineating our previous research on miR-31 published in *Biomaterials*. MiR-31 is a negative regulator for osteogenesis, which has been well studied in different cell types by our research team (see [72,109]). In that study, the adipose tissue-derived stem cells were chosen as the candidate first and they were isolated from F344 rats and cultured. As miR-31 had been demonstrated to be down-regulated during osteogenic induction by miR microarray analysis in other reports, we systematically evaluated its function on rat ADSC differentiation towards osteoblasts. MiR-31 was observed to be reduced during ADSCs’ osteoinductive differentiation and the usage of Lenti-as-miR-31 significantly promoted osteogenesis with enhanced SATB2 levels *in vitro*. Furthermore, the anti-miR-31-modified ADSCs via lentiviral vector were applied to repair critical-sized defects (CSDs) in rats combined with another material, the β-tricalcium phosphate (β-TCP) scaffolds. Micro-CT displayed that miR-31 knockout can improve ossification *in vivo*, albeit, only slight new bone formation was detected in the β-TCP groups [126]. Additionally, it is inspiring that we have also succeeded in repairing rat medial orbital wall defects with miR-31 genetically modified BMSCs, which was released in a recent article [127]. To sum up, our work shed light on the possibility of the application of miRNA-based method to repair CSDs in rats, though the results are merely preclinical data. Yet, it heightens our confidence and encourages us to continue to explore bone defect repair in this fascinating aspect.

**Table 2:** MiRNAs in the regulation of osteoblast differentiation.

MiRNA	Target Gene	Osteogenesis	Cell Line	Reference
let-7f	*Axin2*	+	hBMSC	[104]
miR-10a	*KLF4*	+	hBMSC	[31]
miR-15b	*Smurf1*	+	hBMSC	[39]
miR-20a	*PPARγ Bambi Crim1*	+	hBMSC	[116]
miR-23a	*Runx2*	−	MC3T3	[50]
miR-23a~27a~24-2	*SATB2*	−	MC3T3	[107]
miR-26a	*Smad-1*	−	hADSC	[90]
miR-27	*APC*	+	hFOB1.19	[100]
	*SFRP1*		hFOB1.19	[101]
miR-29a	*osteonectin*	+	MC3T3	[75]
	*DKK1 Kremen2 SFRP2*		hFOB1.19	[102]
	*HDAC4*		MC3T3	[99]
miR-29b	*AcvR2a CTNNBIP1 DUSP2 TGF-β3 HDAC4*	+	MC3T3	[29]
miR-29c	*osteonectin*	+	MC3T3	[75]
miR-30c	*Runx2*	−	MC3T3	[50]
miR-31	*Osx*	−	hBMSC	[32]
	*SATB2*		hADSC	[72]
			rat BMSC	[109]
			rat ADSC	[126]
miR-34a	*JAG1*	−	hBMSC	[33]
miR-34b	*SATB2*	−	primary mouse osteoblasts	[115]
miR-34c	*SATB2*	−	primary mouse osteoblasts	[115]
	*Runx2*		MC3T3	[50]
miR-93	*Osx*	−	primary mouse osteoblasts	[41]
miR-103a	*Runx2*	−	hFOB1.19	[50]
miR-125b	*Osx*	−	hCASMC	[64]
			hBMSC	[65]
	*Smad-4*		hADSC	[91]
	*Cbfβ*		C3H10T1/2	[68]
miR-133	*Runx2*	−	C2C12	[28]
	**		MC3T3	[50]
miR-135a	*Smad-5*	−	C2C12	[28]
	*Runx2*		MC3T3	[50]
miR-137	*Runx2*	−	MC3T3	[50]
miR-138	*FAK*	−	hBMSC	[17]
miR-140-5p	*BMP-2*	−	hBMSC hADCS hUCSC	[22]
miR-141	*Dlx5*	−	MC3T3	[69]
miR-143	*Osx*	−	MC3T3	[58]
miR-145	*Osx*	−	C2C12 MC3T3	[62]
miR-146a	*JMJD3*	−	hUCSC	[74]
	*Smad-2 Smad-3*	+	hSSC	[92]
miR-181a	*TgfbI TβR-I Rgs4 Gata6*	+	MC3T3	[34]
miR-196a	*HOXC8*	+	hADSC	[37]
miR-199a	*Smad-1*	−	C3H10T1/2	[88]
miR-200a	*Dlx5*	‑	MC3T3	[69]
miR-206	*Connexin43*	−	C2C12	[27]
miR-204	*Runx2*	−	ST2 hMSC	[18]
	**		MC3T3	[50]
miR-205	*Runx2*	−	MC3T3	[50]
miR-210	*AcvR1b*	+	ST2	[35]
miR-211	*Runx2*	−	ST2 hMSC	[18]
miR-214	*Osx*	−	C2C12	[63]
	*ATF4*		C2C12	[70]
miR-217	*Runx2*	−	MC3T3	[50]
miR-218	*SOST DKK2 SFRP2*	+	MC3T3	[103]
	*DKK2 SFRP2*		hADSC	[36]
	*Runx2*	−	hDSC	[53]
miR-335-5p	*DKK1*	+	C3H10T1/2	[19]
miR-338	*Runx2*	−	MC3T3	[50]
miR-338-3p	*Runx2 Fgfr2*	−	mouse BMSC	[42]
miR-346	*GSK-3β*	+	hBMSC	[38]
miR-378		+	C2C12	[76]
miR-433	*Runx2*	−	C3H10T1/2	[44]
miR-542-3p	*BMP-7*	−	primary mouse osteoblasts	[89]
miR-548d-5p	*PPARγ*	+	hBMSC	[117]
miR-637	*Osx*	−	hBMSC	[66]
miR-654-5p	*BMP-2*	−	hBMSC	[93]
miR-2861	*HDAC5*	+	ST2	[20]
miR-3960	*HOXA2*	+	ST2	[21]

+: positive regulation; −: negative regulation.

**Table 3:** MiRNAs in the regulation of osteo-related signaling pathways.

MiRNA	Target Component	Regulation on Signaling	Reference
BMP signaling			
miR-26a	Smad-1	−	[90]
miR-29b	TGF-β3 AcvR2a	+	[29]
miR-125b	Smad-4	−	[91]
miR-135a	Smad-5	−	[28]
miR-140-5p	BMP-2	−	[22]
miR-146a	Smad-2 Smad-3	+	[92]
miR-181a	TgfbI TβR-I	+	[34]
miR-199a	Smad-1	−	[88]
miR-210	AcvR1b	+	[35]
miR-542-3p	BMP-7	−	[89]
miR-654-5p	BMP-2	−	[93]
Wnt signaling			
let-7f	Axin2	+	[104]
miR-27	APC SFRP1	+	[100]
miR-29a	HDAC4 DKK1 Kremen2 SFRP2	+	[99,102]
miR-29b	HDAC4 CTNNBIP1	+	[29]
miR-218	SOST DKK2 SFRP2	+	[36,103]
miR-335-5p	DKK1	+	[19]
miR-346	GSK-3β	+	[38]
Notch signaling			
miR-34c	Notch1 Notch2 Jag1	−	[106]
MAPK signaling			
miR-29b	DUSP2	+	[29]

+: positive regulation; −: negative regulation.

## 6. Conclusions

It has been a long time since scientists were fully cognizant of the idea that miRNAs are not an oddity invented by worms to regulate gene expression at post-transcriptional levels. Hundreds of miRNAs are expressed in cells of different species, where they aid in modulating gene expression by mediating mRNA transcript cleavage and/or regulation of translation rate. A great deal of progress has been made in recent years towards the identification and characterization of the miRNAs that regulate gene expression in bone tissues. Perceiving the precise way they exert the multiple biological functions on their diverse targets during MSC differentiation into osteoblasts will facilitate our accurate manipulation of miRNA based stem-cell-mediated bone regeneration. It is believed that miRNAs play a promising role in tissue engineering in the future.
